# Supplementary material for: Maternal and Neonatal Characteristics and Outcomes of COVID-19 in Pregnancy: An Overview of Systematic Reviews
Source: Int J Environ Res Public Health. 2021 Jan 12;18(2):596. doi: 10.3390/ijerph18020596 (PMC7828126; doi:10.3390/ijerph18020596)
Supplement: Supplementary file 1 [file ijerph-18-00596-s001.zip › Supplementary table 1.pdf]

Supplementary table 1: PECOS strategy: category, definition, and search terms in databases.

Category	Definition	Search terms for PubMed	Search terms for Scopus	Search terms for Cochrane Database of Systematic Reviews
Population	Pregnant or recently pregnant (post-partum/ post-abortion/ post-miscarriage) women and their neonates	“Neonatal outcom*” OR “Neonatal characteristic*” OR “Maternal outcom*” OR “Maternal characteristic*” OR “Pregnancy outcom*”	“Neonatal outcom*” OR “Neonatal characteristic*” OR “Maternal outcom*” OR “Maternal characteristic*” OR “Pregnancy outcom*”	“Neonatal outcom*” OR “Neonatal characteristic*” OR “Maternal outcom*” OR “Maternal characteristic*” OR “Pregnancy outcom*”
Exposure	PCR-confirmed or suspected (clinically and radiologically) COVID-19	Covid-19 OR SARS-CoV-2 OR "Coronavirus disease 19"	Covid-19 OR SARS-CoV-2 OR "Coronavirus disease 19"	Covid-19 OR SARS-CoV-2 OR "Coronavirus disease 19"
Comparison	Non-applicable			
Outcomes	Maternal pregnancy and COVID-19 - related outcomes, fetal and neonatal outcomes	“Neonatal outcom*” OR “Neonatal characteristic*” OR “Maternal outcom*” OR “Maternal characteristic*” OR “Pregnancy outcom*” OR “Vertical transmission"	“Neonatal outcom*” OR “Neonatal characteristic*” OR “Maternal outcom*” OR “Maternal characteristic*” OR “Pregnancy outcom*” OR “Vertical transmission"	“Neonatal outcom*” OR “Neonatal characteristic*” OR “Maternal outcom*” OR “Maternal characteristic*” OR “Pregnancy outcom*” OR “Vertical transmission"
Studies	Systematic reviews			

PECOS: population/exposure/comparison/outcomes/studies
