# Supplementary material for: Maternal and Neonatal Characteristics and Outcomes of COVID-19 in Pregnancy: An Overview of Systematic Reviews
Source: Int J Environ Res Public Health. 2021 Jan 12;18(2):596. doi: 10.3390/ijerph18020596 (PMC7828126; doi:10.3390/ijerph18020596)
Supplement: Supplementary file 1 [file ijerph-18-00596-s001.zip › Supplementary table 2.pdf]

FIRST AUTHOR	AIM OF STUDY	DATABASE	DATE OF LAST SEARCH	OUTCOMES	STUDIES TO INCLUDE	PATIENTS TO INCLUDE	NUMBER AND TYPE OF INCLUDED STUDIES	NUMBER OF PARTICIPANTS	CONCLUSIONS	QUALITY ASSESSMENT	META-ANALYSIS CONDUCTED	AMSTAR QUALITY
Diriba et al. [14]	To assess the effect of infection SARS-CoV-2, MERS-CoV, and SARS-CoV infection during pregnancy and its possibility of vertical transmission	PubMed, Web of Science, Embase, Google Scholar and the Cochrane Library	April 30, 2020	Primary outcomes: pregnancy outcomes, secondary outcomes: perinatal outcomes (also mentioned clinical manifestations and maternal outcomes)	No limitations	Pregnant women with confirmed SARS-CoV-2, MERS-CoV, SARS-CoV and other related illness with different clinical features	39	1316 pregnant women, (1271 with SARS-CoV-2, 12 with MERS-CoV and 33 with SARS-CoV)	Coronavirus infection is more likely to affect pregnant women. Respiratory infectious diseases have demonstrated an increased risk of adverse maternal obstetrical complications than the general population due to physiological changes occurred during pregnancy. None of the studies reported transmission of CoV from the mother to the fetus in utero, which may be due to a very low expression of angiotensin-converting enzyme-2 in early maternal–fetal interface cells.	Yes, with binomial distribution formula	Yes	Low
Dhir et al. [15]	To synthesize the available literature on modes of transmission, clinical features and outcomes of SARS-CoV-2 infection in neonates	MEDLINE, EMBASE and Web of Science	June 9, 2020	Perinatal characteristics, clinical features and outcome of RT-PCR proven SARS-CoV-2 infection in neonates	No limitations	Neonates and/or pregnant mothers and reporting data on COVID-19 testing of the neonates	86 (45 case series and 41 case reports)	1992 pregnant women with COVID-19 ranging from 5 to 41 weeks (from case series) and 43 mother-baby dyads (from case reports)	The limited low-quality evidence suggests an extremely low risk of SARS-CoV-2 infections in neonates. Unlike children most of the neonates with proven SARS-CoV-2 infection were symptomatic, and a significant proportion of them required intensive care. Postpartum infection is the commonest mode of acquisition in neonates, although a few cases of congenitally acquired infection are also reported.	Yes, with Newcastle Ottawa Scale	No	Low
Chi et al. [16]	To investigate the outcomes of neonates born to mothers with COVID-19	PubMed/Medline, EMBASE, Cumulative Index to Nursing and Allied Health Literature, National Digital Library of Theses and Dissertations in Taiwan database, Art Image Indexing Service on the Internet Database (Chinese database), and the Cochrane database	March 31, 2020	Primary outcomes: neonatal clinical outcomes and secondary outcomes: infectious status	No limitations	Pregnant women with COVID-19 and their neonates	14	107 pregnant women (3 had not delivered at the publication time of the original study)	Most neonates born to infected mothers had favorable outcomes. Although direct evidences of intrauterine infection were scarce, the risk of intrauterine infection should be considered based on a positive test in 8.8% of the neonates. Symptomatic neonates born to infected mothers should receive tests for SARS-CoV-2 to initiate appropriate treatment and quarantine. Further studies are warranted to assess the outcomes of COVID-19 in neonates.	Yes	Yes	Critically low

Pettirosso et al. [17]	To describe the current understanding of COVID-19 illness in pregnant women and obstetric outcomes	Medline Ovid, EMBASE, World Health Organization COVID-19 research database and Cochrane COVID-19	May 23, 2020	Maternal,obstetric,neonatal outcomes, vertical transmission	Case series, cohort studies	Pregnant women with COVID-19	60	1287 pregnant women	SARS-CoV-2 infection in pregnancy was often asymptomatic. Severe and critical disease rates approximate those in the general population. Vertical transmission is possible; however, it is unclear whether SARS-CoV-2 positive neonates were infected in utero, intrapartum or postpartum. Future work should assess risks of congenital syndromes and adverse perinatal outcomes where infection occurs in early and mid-pregnancy.	No	No	Critically low
Kim et al. [18]	To estimate the CFR of pregnant women infected with SARS-CoV-2 admitted to the intensive care unit	MEDLINE, Embase, and CINAHL	June 4, 2020	Maternal outcomes	English-language case reports, case series, retrospective studies, systematic reviews, and metaanalyses	Pregnant women in at least the second trimester with confirmed COVID-19 admitted in the ICU	15	85 pregnant women admitted to the ICU	The CFR of pregnant women in this report was much lower even if the report from Iran was included. However, this does not take into account the fact that, in general, nonpregnant patients who were critically ill tended to be older and of male gender and have comorbid conditions compared with women of reproductive age.The CFR observed in critically ill pregnant women remains highly concerning. Knowledge of the maternal course of the disease and the degree of increased risk associated with pregnancy is vital in determining management of pregnant women with COVID-19, especially as we prepare for a potential second wave of infections.	No	Yes	Critically low
Gao et al. [19]	To review the clinical features and outcomes of pregnant women with COVID-19	PubMed, Web of Science, EMBASE and MEDLINE	April 16, 2020	Clinical features and pregnancy outcomes of pregnant women with COVID-19	Case reports, case series and observational studies	Pregnant women with COVID-19	14 retrospective case analyses	236 pregnant women with laboratory confirmed COVID-19	The incidences of fever, cough and positive CT findings in pregnant women with COVID-19 are less than those in the normal population with COVID-19, but the rate of preterm labor is higher among pregnant with COVID-19 than among normal pregnant women. There is currently no evidence that COVID-19 can spread through vertical transmission	Yes	Yes	Low
Galang et al. [20]	To summarize clinical presentation, course of illness, and pregnancy and neonatal outcomes in SARS-CoV-1, MERS-CoV, SARS-CoV-2	MEDLINE and ClinicalTrials.gov	April 23, 2020.	Course of illness, indicators of severe illness, maternal health outcomes, and pregnancy outcomes	Articles reporting case-level data on MERS-CoV, SARS-CoV, or SARS-COV-2 infection in pregnant women	Pregnant women with MERS, SARS-COV or SARS-COV-2	46 publications, 12 about MERS-CoV, 7 about SARS-CoV, 31 about SARS-CoV-2	127 pregnant women (98 cases of SARS-CoV-2 infection, 12 with MERS-CoV, 17 with SARS-CoV)	Understanding whether pregnant women may be at risk for adverse maternal and neonatal outcomes from severe coronavirus infections is imperative. Data from case reports of SARS-CoV, MERS-CoV, and SAR-CoV-2 infections during pregnancy are limited, but they may guide early public health actions and clinical decision-making for COVID-19 until more rigorous and systematically collected data are available. The capture of critical data is needed to better define how this infection affects pregnant women and neonates.	No	No	Critically low

Huntley et al. [21]	To ascertain the frequency of maternal and neonatal complications, as well as maternal disease severity, in pregnancies affected by SARS-CoV-2 infection	MEDLINE, Ovid, ClinicalTrials.gov, MedRxiv, and Scopus	April 29, 2020	Primary: maternal ICU admission , critical disease, and death. Secondary: rate of preterm birth, cesarean delivery, vertical transmission, and neonatal death	Case series of at least 10 pregnant patients who tested positive for SARS-CoV-2 infection	Pregnant patients who tested positive for SARS-CoV-2 infection	13 case series	538 pregnant women (462 were laboratory-confirmed)	There are low rates of maternal and neonatal mortality and vertical transmission with SARS-CoV-2. The preterm birth rate of 20% and the cesarean delivery rate exceeding 80% seems related to geographic practice patterns.	Yes, using the methodologic quality and synthesis of case series and case reports described by Murad et al	No	Low
Akhtar et al. [22]	To review published studies related to the association of SARS-CoV-2 infections with pregnancy, fetal, and neonatal outcomes during COVID-19 pandemic	PubMed, Scopus, Medline, Cochrane database, and Google Scholar	May 22, 2020	Pregnancy, foetal, and neonatal outcomes	Cohort studies	Pregnant women with COVID-19	22	156 pregnant women and 108 newborns	COVID-19 infection in pregnancy leads to increased risk in pregnancy complications such as preterm birth, PPROM, and may possibly lead to maternal death in rare cases. There is no evidence to support vertical transmission of SARS-CoV-2 infection to the unborn child. Due to a paucity of inconsistent data regarding the impact of COVID-19 on the newborn, caution should be undertaken to further investigate and monitor possible infection in the neonates born to COVID-19-infected mothers.	Yes with the Newcastle-Ottawa scale	No	Low
Turan et al. [23]	To summarize clinical characteristics and outcomes among pregnant women hospitalized with COVID-19	PubMed, Ovid Medline, Web of Science, and China Academic Literature Database	May 29, 2020	Clinical characteristics., maternal, fetal, neonatal outcomes	Case series, case reports	Pregnant women were admitted to hospital with laboratory-confirmed SARS-CoV-2 infection on RT-PCR tests	63 observational studies, 28 case series, 31 case reports, and 4 retrospective cohort studies	637 pregnant women	Advanced gestation, maternal age, obesity, diabetes mellitus, and a combination of elevated D-dimer and interleukin-6 levels are predictive of poor pregnancy outcomes in COVID-19. The rate of iatrogenic preterm birth and cesarean delivery is high; vertical transmission may be possible but has not been proved.	Yes, using National Institutes of Health quality assessment tools	No	Low
Dubey et al. [24]	To estimate the adverse maternal and neonatal characteristics and outcomes among COVID-19 infected women and determine heterogeneity in the estimates and associated factors	PubMed	July 8, 2020	Adverse maternal and neonatal characteristics, clinical symptoms, pregnancy outcomes. Defined as primary outcomes: C-section rates, preterm birth rates, low birth weight rates, adverse pregnancy events, common symptoms,	No limitations	Pregnant women with COVID-19 and their neonates	61 case series and case reports	790 COVID-19 positive females and 548 neonates	Adverse pregnancy outcomes were prevalent in COVID-19 infected females and varied by location, type, and size of the studies. Regular screening and early detection of COVID-19 in pregnant women may provide more favorable outcomes.	Yes, The risk of bias was assessed using the quality assessment tool for case series studies (NHLBI, Research Triangle Institute International. National Heart, Lung, and Blood Institute Quality Appraisal Tools)	Yes	Moderate

				common treatment								
Capobianco et al. [25]	To assess the risk of clinical complications in pregnant women and neonates infected with SARS-CoV-2	PubMed and Scopus	April 15, 2020	Maternal outcomes, clinical,characteristics, neonatal outcomes, therapy plans, complications in pregnant women with COVID-19 and their neonates	Case-report, case-series, cross-sectional, case-control, and cohort (both prospective and retrospective).	Pregnant women with COVID-19 and their neonates	13 studies conducted in China	114 positive pregnant women	The present study suggests a high rate of maternal and neonatal complications in infected individuals	Yes	Yes	High
Trocado et al. [26]	To evaluate the impact of COVID-19 during pregnancy	PubMed, Scopus database and WHO database	March 20, 2020	Maternal, obstetric and neonatal outcomes	No limitations	Pregnant women with COVID-19	8 (1 was a cohort, 2 were case reports and the remaining 5 were case series)	95 pregnant women and 51 neonates (in the cohort study there were also 41 non-pregnant women and 4 children)	-	Yes, with the Consensus-based Clinical Case Reporting Guideline Development (CARE) checklist	No	Moderate
Khalil et al. [27]	To systematically review the available literature on COVID-19 and pregnancy, to provide comprehensive data and to inform care-providers, pregnant women and their families	Medline, Embase, Clinicaltrials.gov and Cochrane Library	June 8, 2020	Maternal: clinical and laboratory findings, maternal complications, and treatment received, obstetric: preterm birth, stillbirth, mode of delivery and fetal distress, perinatal: perinatal death and evidence of vertical transmission	Case reports, case series, cohort, case-control studies and randomised controlled trials	Pregnant women with PCR confirmed SARS-CoV-2 infection	86	2567 pregnant women	The risk of iatrogenic preterm birth and caesarean delivery was increased-Maternal morbidity is similar to that of women of reproductive age.-Vertical transmission of the virus probably occurs, albeit in a small proportion.	Yes,Newcastle-Ottawa Scale (NOS) or modified NOS for case-control, cohort studies or case series	Yes	High

Yoon et al. [28]	To evaluate the clinical manifestations and outcomes of neonates born to women who had COVID-19 during pregnancy	Medline/PubMed and Embase	April 15, 2020	Neonatal outcomes,maternal clinical and laboratory characteristics with pregnancy outcomes	No limitations	Neonates of laboratory-confirmed COVID-19-positive pregnant women	28, 16 case series and 12 case reports(articles that reported more than five cases were classified as case series and those that reported fewer than five cases were classified as case reports)	223 pregnant women and 201 infants	The majority of reported infants showed no clinical abnormalities - SARS-CoV-2 virus was not detected in most of the neonates- Maternal and fetal mortality is lower with COVID-19 than with SARS-CoV-1 and MERS-CoV infection.- However, COVID-19 during pregnancy might cause severe neonatal and maternal morbidity, even death.	No	No	Critically low
Ashraf et al. [29]	To demonstrate the effects of COVID-19 on pregnant women and the possibility of vertical transmission	PubMed, Scopus, Web of Science, Embase, and Scholar	April 14, 2020	Clinical characteristics of COVID-19 in pregnancy, the risk of pregnancy complications, mortality of mothers and their newborns, and the possibility of vertical transmission	Cohort study, randomized clinical trials, case reports, case series, and letters containing case information	Pregnant women diagnosed with COVID-19 who had recently given birth	21 (case reports, case series, letters)	90 pregnant women diagnosed with COVID-19, 92 neonates born to mothers infected with COVID-19	No differences in the clinical characteristics of pregnant women and non-pregnant COVID-19 patients - COVID-19 infection caused higher incidence of fetal distress and premature labor - The possibility of vertical transmission is rare, four COVID-19 positive neonates in this review	Yes, tool proposed by Murad et al.	No	Low
Thomas et al. [30]	To summarize the current evidence on the vertical transmission potential of COVID-19 infection in the third trimester and its effects on the neonate	OVID MEDLINE, EMBASE, and CENTRAL	May 7, 2020	Maternal characteristics: demographic characteristics, clinical characteristics, SARS-CoV-2 testing, symptomatology, pregnancy characteristics: pregnancy complications, mode of delivery, mortality, neonatal characteristics: SARS CoV-2 testing,complications, mortality	No limitations	Women in the third trimester of their pregnancy	18, 7 case reports, 7 retrospective cohort studies, 1 prospective comparative cohort study, 2 case series, and 1 case control study	157 pregnant patients and 160 neonates	Vertical transmission of SARS-CoV-2 from mother to neonate did not occur	Yes, tool from the CLARITY group at McMaster University for case control and cohort studies, the Joanna Briggs critical appraisal checklist for case series and not assessed for case reports	No	Low

Juan et al. [31]	To evaluate the effects of COVID-19 on maternal, perinatal and neonatal outcomes	PubMed, EMBASE, the Cochrane Library, China National Knowledge Infrastructure Database and Wan Fang Data	April 20, 2020	Maternal and perinatal characteristics, and the clinical manifestations of COVID-19 at admission, including laboratory testing, treatment received and maternal, perinatal and neonatal outcome	No limitations	Laboratory-confirmed and/or clinically diagnosed COVID-19 patient being pregnant on admission	24, 9 case series, 15 case reports	324 pregnant women with COVID-19	Data have insufficient quality to draw unbiased conclusions with regard to the severity of the disease or specific complications of COVID-19 in pregnant women, as well as vertical transmission, perinatal and neonatal complications.	Yes, the Joanna Briggs Institute (JBI) tool for case series and case reports	No	Moderate
Trippella et al. [32]	To provide an overview of the available data on clinical features, outcomes, and management of pregnant women with COVID-19	MEDLINE, EMBASE, Google Scholar	April 18, 2020	Maternal characteristics and outcomes, neonatal characteristics and outcomes	No limitations	Pregnant women and/or neonates, in which a diagnosis of COVID-19 was made with specified diagnostic criteria	37, 19 case reports, 18 case series	275 pregnant women affected with COVID-19 and 248 neonates	Pregnant women with COVID-19 mostly presented with mild or moderate symptoms, with a low incidence of serious complications and adverse outcomes - The outcome of neonates appeared mostly favorable-Despite having a big population sample, the information often derived from low-quality studies (case reports or case series) Hence, although the data are reassuring, they must be confirmed by larger and high-quality studies - Vertical transmission of SARS-CoV-2 was not detected in the majority of the reported cases	Yes, The Joanna Briggs Institute (JBI) Critical Appraisal Checklist	No	Moderate
Walker et al. [33]	To estimate the risk of the neonate becoming infected with SARS-CoV-2 by mode of delivery, type of infant feeding and mother-infant interaction	MEDLINE, Embase and Maternity and Infant Care Database	June 5, 2020	Mode of delivery and neonatal outcomes, breastfeeding and neonatal outcomes, isolation of neonate and COVID-19 infection status in the neonate	Case reports or case series	Pregnant women with confirmed COVID-19 infection, either based on a positive swab, or on high clinical suspicion of COVID-19	49	666 neonates and 655 women	Neonatal COVID-19 infection is uncommon, rarely symptomatic, and the rate of infection is no greater when the baby is born vaginally, breastfed or remains with the mother	Yes, the representativeness of the included mothers was judjed to three populations of women: all pregnant women with SARS-CoV2, all pregnant women with COVID-19 (i.e. symptomatic), all pregnant patients with COVID-19 admitted to hospital.Also the representativeness of the reported babies was judged to the populations of all babies born to women with Covid-19	No	Critically low

de Sousa et al. [34]	To assess the potential risks of COVID-19 infection among pregnant women and consequent fetal transmission	PubMed, Scopus, Embase, ScienceDirect, Web of Science, Google Scholar, bioRxiv and medRxiv	May 26, 2020	Characteristics of pregnant women and newborns, with social, demographic, and clinical data	Observational epidemiological studies and case reports	Pregnant women with COVID-19 infection	49, 21 case reports , 19 cross-sectional descriptive studies, 7 cross-sectional analytical studies, 1 case–control study, 1 cohort study	755 pregnant women and 598 infants	Potential worsening of the clinical conditions of pregnant women infected with SARS-CoV-2 cannot be ruled out, whether the infection is associated with comorbidities or not, due to the occurrence of respiratory disorders, cardiac rhythm disturbances, and acid-base imbalance, among others.	Yes- GRADE system	No	Critically low
Smith et al. [35]	To systematically evaluate the literature and report the maternal and neonatal outcomes associated with COVID-19	PubMed, MEDLINE, and EMBASE	March 28, 2020	Pregnancy measures and neonatal outcomes	No limitations	Pregnant women who were positive for COVID-19	9, 1 retrospective case review, 5 case reports, 3 case series	92 pregnant women	COVID-19-positive pregnant women present with fewer symptoms than the general population and may be RT-PCR negative despite having signs of viral pneumonia. -The incidence of preterm births, low birth weight, C-section, NICU admission appear higher than the general population	Yes, Integrated quality Criteria for Review Of Multiple Study designs (ICROMS) tool	No	Moderate
Kasraeian et al. [36]	To determine the overall effects of COVID-19 pneumonia on positive pregnant women and their newborn infants	PubMed, Google Scholar, MedRXiv, and UpToDate	March 18, 2020	Maternal considerations, fetal considerations and neonatal considerations	No limitations	SARS-CoV-2 positive pregnant woman defined as a patient suffering from COVID-19 pneumonia with a positive lab test of either oropharyngeal swab specimen or polymerase chain reaction	9	87 SARS-CoV-2 positive pregnant women and the 86 newborn infants	No evidence of vertical transmission has been suggested at least in late pregnancy- No hazards have been detected for fetuses or neonates- Most patients suffered from mild or moderate COVID-19 pneumonia with no pregnancy loss, proposing a similar pattern to that of other adult populations.	No	Yes	Moderate
Yang et al. [37]	To summarize available evidence on vertical transmission of SARS-CoV-2	PubMed, the China National Knowledge Infrastructure, CBMdisc, and Wanfang Data	April 20, 2020	Evidence of SARS-CoV-2 infection, evidence of intrauterine vertical transmission	Observational studies or case report/series	Neonates of mothers diagnosed with COVID-19 by real-time polymerase chain reaction (RT-PCR)	22	83 neonates	No direct evidence to support intrauterine vertical transmission of SARS-CoV-2. - No positive RT-PCR results of tests of amniotic fluid, placenta, cord blood, or breast milk have been reported - For women infected during their first and second trimesters, further studies are needed	Yes, Newcastle-Ottawa scale for cohort and case–control studies and a modified tool for case report/series	No	Low
Mustafa et al. [38]	To analyze the disease characterisation in paediatric age group including the possibility of vertical transmission to the neonates	PubMed and Google Scholar	April 2, 2020	Vertical transmission and the outcome of neonates born to COVID19 confirmed mothers, COVID-19 infection in	No limitations	Pregnant women with COVID-19 and their neonates, paediatric patients with COVID-19	33	57 pregnant women, 58 neonates, 251 children and neonates	The disease trajectory in Paediatric patients has good prognosis compared to adults -ICU and death are rare - Vertical transmission and virus shedding in breast milk are yet to be established	No	Yes	Critically low

				children and neonates							
Parazzini et al. [39]	To review the available information on mode of delivery, vertical transmission, and neonatal outcome in pregnant women with COVID-19	Embase and PubMed	March 31, 2020	Primary outcomes: frequency of preterm birth (<37 weeks of gestation), vaginal delivery, Apgar score at 5 minutes <7, and newborn infection	Case reports/observational studies	Women treated for SARS-CoV-2 infection	13, 6 case reports, 7 retrospective clinical series	64 pregnant women who delivered	The rate of vertical or peripartum transmission of SARS-CoV-2 is low, if any, for cesarean delivery-No data are available for vaginal delivery-Low frequency of spontaneous preterm birth and general favorable immediate neonatal outcome are reassuring	No	No	Critically low
Allotey et al. [40]	To determine the clinical manifestations, risk factors, and maternal and perinatal outcomes in pregnant and recently pregnant women with suspected or confirmed COVID-19	Medline, Embase, Cochrane database, WHO COVID-19 database, CNKI, Wanfang, LOVE	June 26, 2020	Rates of covid-19, clinical manifestations, risk factors and maternal and perinatal outcomes in women with covid-19	Primary case reports, case series, observational studies or randomised-controlled trials.	Pregnant, postpartum and post abortion/miscarriage women with suspected or confirmed COVID-19 infection.	77 cohort studies (55 comparative, 22 non-comparative)	13118 pregnant and recently pregnant women with covid-19; 83486 women of reproductive age with covid-19	Pregnant and recently pregnant women are less likely to manifest covid-19 related symptoms of fever and myalgia than non-pregnant women of reproductive age and are potentially more likely to need intensive care treatment Pre-existing comorbidities, high maternal age, and high body mass index seem to be risk factors for severe covid-19. Preterm birth rates are high in pregnant women with covid-19 than in pregnant women without the disease.	Yes, Newcastle-Ottawa scale	Yes	Moderate
Yang Z et al. [41]	To summarize available evidence on maternal, fetal, and neonatal outcomes of pregnant women with COVID-19	PubMed, Google Scholar, CNKI, Wanfang, VIP, and CBMdisc	March 26, 2020	Maternal, fetal, and neonatal outcomes of pregnant women	Observational studies, case reports/series, letters, and comments - no restriction on language	Pregnant women with COVID-19	18 studies (17 case reports/series and 1 case-control study)	114	clinical characteristics of pregnant women with COVID-19 similar to those of non-pregnant adults - fetal and neonatal outcomes good in most cases - available data only on pregnant women infected in their third trimesters - further studies on long-term outcomes and potential intrauterine vertical transmission are needed	Yes - Newcastle-Ottawa Scale and a modified tool for quality appraisal of case reports/series	No	Low

Mullins et al. / rapid review [42]	To guide management of women affected by COVID-19 during pregnancy, which was used to develop the RCOG guidelines	PubMed and MedRxiv	March 10, 2020	Maternal outcome, early pregnancy outcomes, second/third-trimester pregnancy loss, prematurity, fetal growth and placental effects, delivery and postnatal outcomes and neonatal outcomes for CoV spectrum infections	Primary case reports, case series and randomized controlled trials - no date or language restrictions	Women of any age affected by coronavirus in pregnancy or the postnatal period	21	32	Compared with SARS and MERS, COVID-19 appears less lethal - Preterm delivery affected 47% of women hospitalized with COVID-19	Yes - Subjective/anecdotal assessment as low, medium, high	No	Critically low
Zaigham et al. [43]	To summarize the clinical manifestations and maternal and perinatal outcomes of COVID-19 during pregnancy	MEDLINE, Embase and Google Scholar	April 4, 2020	Clinical manifestations and maternal and perinatal outcomes	English or Chinese, availability of clinical characteristics including maternal and perinatal outcomes - No limitation in study type	Laboratory-confirmed COVID-19 infection using quantitative real-time polymerase chain reaction (qRT-PCR) or dual fluorescence polymerase chain reaction (PCR), patient pregnant on admission	18 (14 case reports, 4 case series)	108 pregn	Majority of mothers without any major complications - severe maternal morbidity as a result of COVID-19 and perinatal deaths were reported -Vertical transmission of the COVID-19 not ruled out	Yes - framework for appraisal, synthesis and application of evidence suggested by Murad et al - based on the domains of selection, ascertainment, causality and reporting	No	Moderate

Della Gatta et al. [44]	To collect the available information about the impact of COVID-19 on mothers and neonates and to focus on time and mode of delivery	PubMed, CINAHL, and Scopus	March 16, 2020	Clinical features, symptoms, associated diseases, fetal characteristics, time of delivery, type of delivery, and follow-up	Reports of pregnancies' management complicated by COVID-19 - no restriction on language (Chinese and English) - only studies in which the diagnosis was based on the criteria provided by the New Coronavirus Pneumonia Prevention and Control Program (4th edition and subsequent editions) / study type published by the National Health Commission of China	Pregnant patients aged 20 year or older	6 studies (retrospective: 1 case report, 5 case series one with control group)	51 (1 not laboratory confirmed)	High rate of preterm delivery by cesarean delivery is a reason for concern. Cesarean delivery was typically an elective surgical intervention, and it is reasonable to question whether cesarean delivery for pregnant patients with coronavirus disease 2019 was warranted	Yes - methodological quality of case reports and case series described by Murad et al	No	Low

Segars et al. [45]	To summarize understanding of the effects of novel and prior coronaviruses on human reproduction and in pregnancy.	PubMed and Embase	April 6, 2020	Reproductive outcomes, effects on gametes, pregnancy outcomes, and neonatal complications	English, both published and peer-reviewed on-line publications - no limitation on sample size - all reports including pregnancy or reproductive tissues were included - excluded editorials and publications of guidelines, duplicates or did not contain information related to pregnancy or reproduction, the presence of virus in reproductive tissues, effects on gametes, pregnancy outcomes, or neonatal complications	Pregnant COVID-19 patients and/or their neonates	19 case reports or series and two case-control reports	162, 184 infants	Coronavirus Disease 2019 infection may affect adversely some pregnant women and their offspring	No	No	Moderate
Chang et al. [46]	To collect current evidence about COVID-19 in children	PubMed, Embase, manual searches of Google Scholar and the Chinese Medical Journal Network	March 15, 2020	Epidemiological, clinical, laboratory and radiographic features of COVID-19 positive children and demographics and outcomes of neonates born to COVID-19 positive mothers	No limitation if patient associated eligibility criteria were fulfilled	Women younger than 18 years old,COVID-19 positive confirmed with PCR	* all parameters regard our outcome of interest - 2 case series	19	Currently, there is no evidence of vertical transmission to neonates born to mothers with COVID-19 - COVID-19 has distinct features in children. The disease severity is mild. Current diagnosis is based mainly on typical ground glass opacities on chest CT, epidemiological suspicion and contact tracing	Yes - only of studies included in meta-analysis (Newcastle–Ottawa Scale) , NOT in studies of interest		Moderate

Muhidin et al. [47]	To clarify the impact of COVID-19 on pregnant patients and maternal and neonatal outcomes	PubMed, Scopus, Embase, ProQuest, and Science Direct	March 19, 2020	Clinical features diagnosis and treatment, obstetric outcomes, mode of delivery, neonates' outcomes , vertical transmission potential	All studies including original data; case reports, case series, descriptive and observational studies, and randomized controlled trials - articles published in English or Persian language that contained the keywords in their title, abstract or keywords, and their full-text was available	Pregnant women with confirmed/ suspected COVID-19 and their infants	9 (3 case reports, 2 retrospective case series, 3 case series, 1 case control study)	89 pregnant women (68 with confirmed, 18 with suspected, 3 discharged with an uncomplicated ongoing pregnancy) --> 86	Pregnant patients in late pregnancy had clinical manifestations similar to non-pregnant adults. It appears that the risk of fetal distress, preterm delivery and prelabor rupture of membranes (PROM) rises with the onset of COVID-19 in the third trimester of pregnancy. There is also no evidence of intrauterine and transplacental transmission of COVID-19 to the fetus in the third trimester of pregnancies	yes - Quality assessment tools of the National Institutes of Health (NIH) consisting of 9 items for case-report studies and 12 items for case-control studies	No	Moderate
Di Mascio et al. [48]	To report pregnancy and perinatal outcomes of CoV spectrum infections during pregnancy	Medline, Embase, CINAHL, and Clinicaltrials.gov	March 13, 2020	Pregnancy: preterm birth (PTB; either before 37 or 34 weeks of gestation), preeclampsia (PE), preterm prelabor rupture of membranes (pPROM), fetal growth restriction (FGR), miscarriage, c-section, perinatal: fetal distress, Apgar score <7 at 5 minutes, neonatal asphyxia, admission to neonatal intensive care unit (NICU), perinatal death, including both stillbirth and neonatal death, evidence of	English language articles	Hospitalized pregnant women with a confirmed coronavirus-spectrum illness, defined as SARS, MERS or COVID-19 infection	19 total, 6 on COVID-19 (4 retrospective/case series, 2 case reports)	79; 41 (51.9%) were affected by COVID-19	COVID-19 infection was associated with higher rate (and pooled proportions) of preterm birth, preeclampsia, cesarean, and perinatal death. There have been no published cases of clinical evidence of vertical transmission.	Yes - methodological quality and synthesis of case series and case reports described by Murad et al	Yes - MA of proportions - random effects model	High

				vertical transmission								
Shi et al. [49]	To identify the most common laboratory abnormalities in pregnant women with COVID-19	PubMed, CNKI and Wanfang	April 20, 2020	Laboratory data in pregnant women with confirmed COVID-19	No country, race, or language restrictions	Pregnant women with COVID-19	11	173	Elevated D-dimer levels, elevated neutrophil count, elevated C-reactive protein levels, and decreased lymphocyte count are the most prevalent laboratory abnormalities in pregnant women with COVID-19	No	Yes (random effects model)	Moderate
Elshafeey et al. [50]	To summarize the existing literature on COVID-19 infection during pregnancy and childbirth	LitCovid, EBSCO MEDLINE, CENTRAL, CINAHL, Web of Science, and Scopus	April 19, 2020	Clinical presentation of COVID-19 during pregnancy, the spectrum of COVID-19 disease severity during pregnancy, the maternal adverse outcomes in cases of COVID-19, the fetal and neonatal outcomes in	Any article reporting original research of COVID-19 during pregnancy, whether diagnosis was confirmed by reverse-transcription polymerase chain reaction (RT-PCR) or based on clinical,	Pregnant women with COVID-19 whether diagnosis was confirmed by reverse-transcription polymerase chain reaction (RT-PCR) or based on clinical, imaging, and laboratory criteria	33 (1 case-control, 16 case reports, 16 case series)	385 women with suspected/confirmed COVID-19 during pregnancy and childbirth, 346 (89.9%) RT-PCR confirmed, 39 (10.1%) diagnosed based on clinical and radiological features	COVID-19 infection during pregnancy probably has a clinical presentation and severity resembling that in non-pregnant adults. It is probably not associated with poor maternal or perinatal outcomes.	No	No	Critically low

				cases of COVID-19	imaging, and laboratory criteria							
Banaei et al. [51]	To investigate the impact of COVID-19 on obstetrics and neonatal outcomes	PubMed, Web of Science, Scopus, ProQuest, Embase and Google scholar	April 10, 2020	The impact of COVID-19 on obstetrics and neonatal outcomes	All studies that indicate the impact of COVID-19 on obstetrics and neonatal outcomes	Pregnant women with a diagnosis of COVID-19 by using laboratory tests and/or imaging examinations and their neonates	16 studies: 6 retrospective case analyses, 1 cohort, 4 case series and 5 case reports	123 pregnant women with a definitive diagnosis of COVID-19 and their neonates	COVID-19 may cause negative outcomes in both mothers and neonates. However, there were evidence about neonate infected with COVID-19, but there is controversial information about vertical transmission	Yes: Newcastle-Ottawa Scale (NOS) checklist for cross-sectional, case control and cohort studies, checklist by Kanthraj et al for case reports	No	Critically low
Matar et al. [52]	to describe the clinical characteristics and perinatal outcomes of COVID-19 in pregnancy	vid Medline, Epub Ahead of Print, in-Process, other Non-Indexed Citations, Ovid Embase, Ovid Cochrane Central Register of Controlled Trials, and Scopus	April 30, 2020	Maternal clinical characteristics, laboratory and imaging findings and perinatal outcomes	Case series and case reports concerning RT-PCR Covid-19 positive pregnant women that also include report of neonatal outcomes	Pregnant women with RT-PCR nasal or throat swabs positive for SARS-CoV-2	24 studies (10 case reports, 4 case series, 1 case control, 8 retrospective cohorts, 1 prospective cohort)	136	Clinical picture in pregnant women with COVID-19 did not differ from the nonpregnant population - rate of preterm birth and cesarean delivery considerably higher than international averages	Yes: Murad et al	Yes	Moderate
