# Supplementary material for: Maternal and Neonatal Characteristics and Outcomes of COVID-19 in Pregnancy: An Overview of Systematic Reviews
Source: Int J Environ Res Public Health. 2021 Jan 12;18(2):596. doi: 10.3390/ijerph18020596 (PMC7828126; doi:10.3390/ijerph18020596)
Supplement: Supplementary file 1 [file ijerph-18-00596-s001.zip › Supplementary table 3.pdf]

Supplementary table 3.

Abbreviation	Explanation
N	Sample screened for the outcome
n	Number of included participants with the outcome
p	Pooled proportion
PROM	Premature rupture of membranes
pPROM	Preterm premature rupture of membranes
NA	Non-applicable
SOB	Shortness of breath
CRP	C-reactive protein
PCT	Procalcitonin
LFTs	Liver Function Tests
ALT	Alanine aminotransferase
AST	Aspartate aminotransferase
